# Tumour targeting of humanised cross-linked divalent-fab′ antibody fragments: a clinical phase I/II study

**DOI:** 10.1038/sj.bjc.6600198

**Published:** 2002-05-06

**Authors:** J L Casey, M P Napier, D J King, R B Pedley, L C Chaplin, N Weir, L Skelton, A J Green, L D Hope-Stone, G T Yarranton, R H J Begent

**Affiliations:** Cancer Research UK Targeting and Imaging Group, Department of Oncology, Royal Free and University College Medical School, University College London, Royal Free Campus, Rowland Hill Street, London NW3 2PF, UK; Celltech Therapeutics, 216 Bath Road, Slough, Berkshire SL1 4EN, UK

**Keywords:** humanised antibody, divalent-Fab′ maleimide, fragments, hDFM, imaging, colorectal cancer, phase I/II trial

## Abstract

Antibody engineering has made it possible to design antibodies with optimal characteristics for delivery of radionuclides for tumour imaging and therapy. A humanised divalent-Fab′ cross-linked with a bis-maleimide linker referred to as humanised divalent-Fab′ maleimide was produced as a result of this design process. It is a humanised divalent antibody with no Fc, which can be produced in bacteria and has enhanced stability compared with F(ab′)_2_. Here we describe a clinical study in patients with colorectal cancer using humanised divalent-Fab′ maleimide generated from the anti-carcinoembryonic antigen antibody A5B7 radiolabelled with iodine-131. Ten patients received an i.v. injection of iodine-131-labelled A5B7 humanised divalent-Fab′ maleimide, and positive tumour images were obtained by gamma camera imaging in eight patients with known lesions, and one previously undetected lesion was identified. True negative results were obtained in two patients without tumour. Area under the curve analysis of serial blood gamma counting and gamma camera images showed a higher tumour to blood ratio compared to A5B7 mF(ab′)_2_ used previously in the clinic, implying this new molecule may be superior for radioimmunotherapy. MIRD dose calculations showed a relatively high radiation dose to the kidney, which may limit the amount of activity that could be administered in radioimmunotherapy. However the reduction in immunogenicity was also a major advantage for A5B7 humanised divalent-Fab′ maleimide over murine versions of this antibody suggesting that humanised divalent-Fab′ maleimide should be a useful vehicle for repeated therapies.

*British Journal of Cancer* (2002) **86**, 1401–1410. DOI: 10.1038/sj/bjc/6600198 www.bjcancer.com

© 2002 Cancer Research UK

## 

A5B7 is a murine monoclonal antibody raised against carcinoembryonic antigen (CEA). A5B7 IgG and its F(ab′)_2_ fragment have been radiolabelled with ^125^I for radioimmunoguided surgery (RIGS) and ^131^I for radioimmunotherapy (RIT) and radio immunoscintigraphy (RIS) in patients with colorectal carcinoma (Lane *et al*, 1994; Theodorou, 1995). RIT has produced responses in patients after the failure of conventional 5 fluorouracil based chemotherapy treatments (Lane *et al*, 1994). The maximum tolerated dose of ^131^I was 2.2 GBq m^−2^ with myelosuppression being the dose-limiting factor (Lane *et al*, 1994). This study also showed a more efficient early tumour uptake of the F(ab′)_2_ fragment (two-fold increase) compared with the intact IgG.

A major limitation of RIT that has affected most clinical studies is the production of human anti-mouse antibodies (HAMA) to murine immunoglobulins as this often prevents re-treatment. Anti-mouse antibodies form complexes with the tumour specific antibody, which are rapidly cleared from the circulation so preventing tumour localisation. Presently immunosuppressive drugs such as cyclosporin A (CsA) are administered to reduce HAMA formation (Ledermann *et al*, 1988). In the most recent RIT study using A5B7 described by Lane *et al* (1994), oral administration of CsA enabled up to four repeated treatments of ^131^I-A5B7. However, use of immunosuppressive drugs is not favoured due to side effects and incomplete effectiveness.

The development of antibodies with low immunogenicity is therefore important to allow repeated administration in RIT. Technologies to generate human antibodies such as human hybridoma technology, transgenic or SCID-hu mice and recombinant libraries are now available for production of high affinity human antibodies (Borrebaeck *et al*, 1990; Lonberg *et al*, 1994; Winter *et al*, 1994). As an alternative, existing murine antibodies have been reconstructed by grafting the critical portions of the antigen binding regions (CDR's) onto a human antibody framework (Verhoeyen *et al*, 1988). Clinical trials involving several humanised antibodies are currently in various stages of progress (Emery and Adair, 1994), and the results so far indicate that immunogenicity is in fact reduced or completely absent using this approach (Hale and Phillips, 1995; Stephens *et al*, 1995).

Antibody engineering has enabled the design of antibodies with superior properties to whole IgG's for delivery of radionuclides for tumour imaging or therapy. We have recently performed a series of experiments to find the optimal characteristics of the antibody A5B7 for RIT. A5B7 was humanised to reduce immunogenicity and was found to target tumour effectively (Adair *et al*, 1992). In addition DFM was modified to contain a single hinge thiol suitable for chemical cross-linking to form multivalent di-Fab′ and tri-Fab′ molecules (Casey *et al*, 1996). A comparison of the properties of murine A5B7 DFM and TFM with parent F(ab′)_2_ and IgG was performed and kinetic analysis of antigen binding revealed that DFM had a significantly faster association rate than all other antibody forms (Casey *et al*, 1996). This may be a consequence of the increased spacing or flexibility of the chemical cross-linker. Although the biodistribution data demonstrated equivalent tumour uptake levels it was concluded that ^131^I -DFM due to its faster association rate might prove to be superior to ^131^I -F(ab′)_2_ in the clinic. In another study a re-oxidised version of humanised A5B7 hF(ab′)_2_ was compared to chemically cross-linked hDFM; these are identical constructs apart from the bond between the two Fab′ arms. The *in vitro* and *in vivo* stability was analysed and A5B7 hDFM proved to have a higher affinity and a significantly improved tumour uptake to the hF(ab′)_2_ (Casey *et al*, 1995).

Although preclinical studies in nude mouse model systems provide valuable information in selecting the most appropriate form of antibody, pilot human investigations are necessary to assess whether a particular antibody will be clinically useful. In this study a batch of A5B7 hDFM, suitable for clinical purposes, was prepared to assess the safety and tumour targeting in patients with colorectal cancer, with the aim of the possible use of this humanised version of A5B7 for future RIT studies. Bacterial production of hFab′ was facilitated with the use of production scale fermentation, which is becoming an attractive alternative to mammalian expression systems, which are relatively expensive. A5B7 hDFM is one of the first antibodies to be produced in *E. coli* for the clinic.

## MATERIALS AND METHODS

### Production of clinical grade hDFM and mF(ab′)_2_

Clinical grade A5B7 hDFM was produced in accordance to the Cancer Research Campaign (CRC) specified guidelines for production of recombinant proteins for clinical use in the UK (Begent *et al*, 1993). A5B7 murine F(ab′)_2_ was prepared for clinical studies as previously described by Lane *et al* (1994) in accordance with the CRC Operation Manual (1986).

### Fermentation of A5B7 hFab′

A5B7 hFab′ was expressed in *E. coli* strain W3110pMRR45 as previously described (Begent *et al*, 1996). Material was generated at pilot scale using a 200 litre vessel operating in fed batch mode. The defined salts medium SM6B was used for seed and production fermentations, and product formation was induced by switching carbon source from glucose to lactose. Cells were harvested 20 h post induction by continuous centrifugation. Tris/EDTA buffer pH 7.4 was added to the cell slurry to give a final concentration of 50 mM EDTA. The cell suspension was then homogenised to release Fab′ from the cell periplasm and the homogenate heat treated at 46°C for 16 h to remove unassembled Fab′. The extract was then centrifuged to remove cell debris and the centrate clarified using tangential flow filtration on 0.2 μM membranes. The permeate was made to 1 M glycine by the addition of solid glycine and adjusted to pH 7.5 with sodium glycinate solution (50% w v^−1^).

### Purification of A5B7 hFab′

Purification of hFab′ was performed using streamline A® affinity chromatography. Streamline A® matrix (Pharmacia, UK) was conditioned prior to use by washing with 6 M guanidine HCl and equilibrated with 1 M glycine/glycinate, 25 mM NaCl pH 8.0. The matrix was added to the sample as a slurry and allowed to bind overnight at 4°C. The slurry was packed into a column at a flow rate of 200 cm h^−1^ and washed with 10 column volumes (c.v.) of equilibration buffer. The hFab′ was eluted with 2–3 c.v. of 0.1 M tri-sodium citrate/citric acid pH 3.0 at a flow rate of 50 cm h^−1^. The hFab′ containing fractions were pooled, pH adjusted to 5.5 by addition of 2 M tris-HCl pH 8.5 and sterile filtered through a 0.2 μm filter.

### Preparation of cross-linked A5B7 hDFM

Streamline A® purified hFab′ was pooled and concentrated to 10 mg ml^−1^ in an Amicon stirred cell using a 10 kDa molecular weight cut-off membrane, pre-soaked in sterile water. For reduction hFab′ was dialysed into 0.1 M sodium acetate pH 6.0 with 3 buffer changes and incubated with a final (optimised) concentration of 50 mM 2 mercaptoethylamine for 90 min at 37°C with constant agitation. The reductant was removed by buffer exchange into 0.1 M sodium acetate pH 6.0 using a Sephadex G25 column (70 ml: 2.6×13 cm) run at a flow rate of 3.0 ml min^−1^, collecting 2.75 ml fractions. Each fraction was analysed for the presence of free thiol groups by performing a thiol assay as described previously (Casey *et al*, 1996).

Cross-linker 1,6, bismaleimidohexane (Pierce Warriner; UK) was added in five equal aliquots as a 1 mM solution in dimethylformamide to the reduced hFab′ to a final molar ratio of 2.2 : 1 (Fab′: linker) and incubated with constant agitation over 30 min at 37°C. The reaction was then allowed to proceed overnight to form hDFM.

### Purification of hA5B7 DFM

A gel filtration column (5.0×100 cm) was packed with approximately 1.5 litre sephacryl S-200HR and equilibrated with 50 mM sodium phosphate buffer. The cross-linked sample was loaded to the column and run at a flow rate of 5 ml min^−1^ and 2 ml fractions were collected. Relevant fractions containing pure hDFM were pooled, concentrated by Amicon ultrafiltration then applied to an endotoxin removal column as detailed previously by Casey *et al* (1995).

Endotoxin free material was concentrated using Amicon ultrafiltration, 0.2 μm filter sterilised and dispensed in a sterile hood into 0.5 mg aliquots which were stored at 4.0°C until required.

### Characterisation, toxicology and safety testing

Final aliquots of hDFM were fully characterised before and after radiolabelling for stability and immunoreactivity by ELISA and HPLC analysis. A biodistribution experiment with ^131^I-hDFM (0.37 MBq per mouse) was performed to ensure tumour localisation *in vivo* of the patient material using methods described previously (Casey *et al*, 1996). Toxicology (*in vivo*) and safety analysis (pyrogen testing, microbiological and bacterial DNA screening) was performed in accordance with the requirements of the CRC regulatory committee UK (Begent *et al*, 1993).

### Clinical trial design

A series of 10 patients were recruited for a phase I/II single centre open clinical trial of ^131^I-hDFM. Eight had metastatic or recurrent colorectal cancer with tumour CEA production confirmed by raised serum CEA concentration or by positive CEA immunohistochemistry staining of biopsy specimens. Two further patients with suspected colorectal cancer recurrence acted as negative controls. One (patient 7) had a sacral bony metastasis suggested on a technetium^99m^ bone scan but a subsequent biopsy did not confirm tumour recurrence. Another (patient 10) had an elevated tumour marker CA19-9, but subsequent conventional computerised tomography (CT), magnetic resonance (MRI), and positron emission tomography (PET) imaging as well as laparotomy failed to confirm a recurrence. Nineteen patients were recruited for the ^131^I-mF(ab′)_2_ study who had unresectable, locally recurrent or metastatic tumours, most patients had raised CEA levels at the time of recruitment for the study.

All patients required a WHO performance status of 0–2, and gave written informed consent. The study was approved by ethics committee and covered by ARSAC licence. The thyroid was blocked with potassium iodide tablets (50 mg tds) for 10 days beginning the day before the antibody scan, and potassium perchlorate (200 mg qds for 1 day) on the day of administration. A few hours prior to the injection of radiolabelled antibody, patients were given an intradermal skin test injection to observe any adverse allergic reaction to the antibody. The skin test consisted of 10 μg antibody (0.2 ml) and a saline control. Patients were excluded from the study if they showed a positive reaction to the antibody (wheal >5 mm at 15 min).

^131^I-hDFM and mF(ab′)_2_ (0.5 mg) were radiolabelled using the chloramine T method to a specific activity of 0.22–0.37 MBq μg^−1^ and 111–185 MBq was administered to patients using an i.v. cannula, which was flushed with saline before and after radio-antibody infusion. Whole body planar and single photon emission computerised tomography (SPECT) scans of the thorax and abdomen were performed on a Gemini 700 gamma camera at 2, 5, 24, 47 and 72 h (four patients only) post administration of radiolabelled antibody. SPECT images were reconstructed using IGE filtered backprojection software, and corrected for decay, photon attenuation and Compton scatter (Green *et al*, 1990). Scans were analysed independently by two qualified physicians and consensus reporting was performed in the event of a discrepancy. Patients also had conventional confirmatory CT and X-ray scanning or appropriate MRI scanning to confirm results were true positive or negative.

### Pharmacokinetic analysis and dosimetry estimates

To assess the clearance and half-life of ^131^I-hDFM in the circulation samples of patient's urine and whole blood (in 2 ml heparinised tubes) were collected at each scanning time. Blood samples (0.5 ml) were dispensed in triplicate to pre-weighed tubes and re-weighed before counting for ^131^I activity in a gamma counter. All samples were counted together, followed by standards consisting of dilutions of the patient injectate. The percentage of injected activity per kg (% i.a. kg^−1^) was evaluated and the half-life in blood was calculated by fitting data to a bi-exponential model using the least sum of the squares regression method. We analysed urine samples by SDS–PAGE autoradiography and Western blotting to analyse renal clearance of ^131^I-hDFM. Complete 24 h urine collection to 82 h post administration was carried out for one patient to measure the total urinary output of ^131^I-hDFM.

Additional serum and plasma samples were taken at each scanning time for stability, immunogenicity and antigen binding analysis. Stability analysis was performed by HPLC using the method previously described by Stephens *et al* (1995). Plasma samples collected from patients were analysed for ability to bind to antigen. Briefly, 100 μl plasma samples were applied to microtitre wells coated with CEA or phosphate buffer (control) in duplicate and incubated for 1 h at room temperature on a plate shaker. The microtitre plate was washed four times with 50 mM sodium phosphate buffer/0.05%Tween 20 (wash buffer) and four times with dH_2_O. Each well was counted for ^131^I activity in a gamma counter. An ELISA was designed to assess patients' immune response to hDFM, before and at 14 days and 2 months (approximately) after injection of radiolabelled antibody. We measured the human anti-human antibody response (HAHA) since hDFM contains a human antibody framework. Microtitre plates (Maxisorp, Nunc) were coated with 100 μl of a 5 μg ml^−1^ solution of hDFM in 0.2 M sodium carbonate buffer pH 9.6 and incubated at room temperature for 1 h. The plate was blocked overnight with 250 μl 50 mM sodium phosphate buffer/5% BSA then washed four times with wash buffer. A series of dilutions of test serum were prepared and 100 μl per well in duplicate was incubated at room temperature with gentle mixing. The plate was washed as above and incubated with 100 μl goat anti-human Fc IgM or IgG conjugated to horseradish peroxidase (Jacksons Research Labs, USA) at 1/500 or 1/1000 respectively, and incubated for 1 h. After final washing the assay was detected with 3,3′,5,5′-tetramethylbenzidine (TMB, Sigma) substrate.

Dosimetry analysis of tumour and normal tissues was performed by selecting individual 0.88 cm^3^ regions of interest (ROI) taken from SPECT images (Lane *et al*, 1994), with the aid of corresponding CT images. These values were fitted to a bi-exponential clearance model to estimate the total residence time or area under the curve (AUC). Values were corrected for β dose radiation (cGy) to each tissue using the S factor for ^131^I (MIRD pamphlet no 11, 1975), and the MIRD absorbed dose weighting system (ICRP 60, 1990) which corrects for cross-organ doses.

## RESULTS

### Production of clinical grade hDFM

Large-scale fermentation of hFab′ resulted in the majority of hFab′ in monomer form. Purification using Streamline A® generated a yield of approximately 85 mg.

Cross-linking resulted in 44% hDFM, with no detectable (<1%) reoxidised F(ab′)_2_. Final purification of hDFM from unreacted hFab′ was performed by sephadex S-200 gel filtration of individual cross-linked fractions. The yield of hDFM after gel filtration was 28.5 mg. This material was passed over the endotoxin removing column twice and analysed for presence of endotoxin. The final yield of concentrated endotoxin free material was 26 mg.

### Characterisation, toxicology and safety testing of clinical grade hDFM

Aliquots of hDFM were retained for characterisation analysis, toxicology and assessment of contaminants. For characterisation hDFM was analysed for purity by analytical gel filtration using HPLC and by SDS–PAGE. A single peak of the correct molecular weight was produced demonstrating >95% of the clinical product was in the form of hDFM.

Immunoreactivity to CEA was assessed by competition ELISA with unmodified IgG conjugated to HRP. Similar binding to a previous batch of hDFM (known to localise to human tumour xenografts) was achieved, suggesting that the patient batch of hDFM was fully immunoreactive. Immunohistochemical analysis revealed binding of hDFM (biotinylated) to CEA producing tumours with similar strength to murine A5B7 (biotinylated), and retention of the high level of specificity and low cross-reactivity observed for the parent antibody.

**Figure 1:**
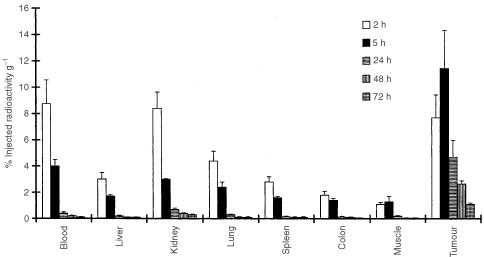
Biodistribution of ^131^I-hDFM (patient batch) in nude mice bearing LS174T human colorectal tumour xenografts. Time-points at 2 h (column 1), 5 h (column 2), 24 h (column 3), 48 h (column 4) and 72 h (column 5) post injection. Results are expressed as per cent injected activity per gram of tissue, columns are a mean of four mice and error bars represent standard deviations.

An aliquot of hDFM was test labelled with 185 MBq^131^I, with >90% incorporation. TLC analysis after desalting confirmed that 98.8% of radiolabel was attached to hDFM. Antigen binding post labelling, measured by applying a sample to a 1 ml CEA column, produced 98% retention of antigen binding post labelling. This was confirmed by performing an ELISA with samples pre and post labelling. Gel filtration analysis after radiolabelling showed the presence of a single peak of molecular weight 100 kDa, with no evidence of aggregation or breakdown. A sample of ^131^I-hDFM was incubated in normal human serum for 24 h (approximately) and analysed by gel filtration. The trace showed a single peak consistent with hDFM. Radiolabelled hDFM in nude mice bearing human colorectal tumour xenografts showed good tumour uptake and retention, with no uncharacteristic binding to other normal organs (Figure 1

).

Toxicology showed there were no signs of disease or fever in guinea pigs following administration of 10× the patient dose of hDFM (unlabelled) over a 28 day observation period. The patient material was free of pyrogens and bacterial DNA. Microbial testing also revealed no evidence of bacterial or fungal growth after incubation for 2 days at 37°C.

### Blood and urine clearance

**Figure 2:**
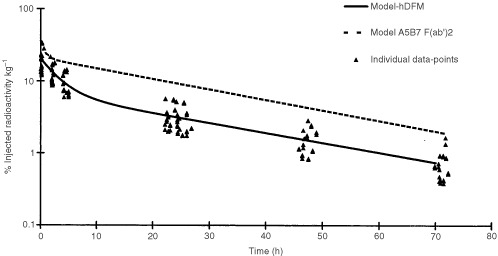
Modelled clearance of hDFM from blood. Blood clearance of the murine A5B7 F(ab')_2_ is charted for comparison. Each diamond reflects an individual data-point from which the model is derived.

Clearance of ^131^I-hDFM from the circulation was monitored in patients by counting regular blood samples (10 min, 2, 4–5, 24, 26, 48 and 72 h) and calculating the per cent injected radioactivity (i.a.) kg^−1^. These data were fitted to a bi-exponential model and the best model fit to the data is illustrated in Figure 2

. The half-lives for α and β phases were calculated for individual patients, the median α-half-life was 2.12 h (range 1.3–3.1 h) and median β-half-life was 20.3 h (range 14–26.3 h). The modelled α and β-half-lives for the whole data set were 2.18 and 22.01 h respectively. This is comparable to the modelled clearance for ^131^I-mF(ab′)_2_ in man with a median α-life of 0.54 h and median β-half-life of 20.7 h, also shown in Figure 2.

Samples of urine were analysed by SDS–PAGE and autoradiography. For most patients there was no evidence of breakdown products in the urine. For three patients, low molecular weight bands in the urine were observed, these consisted of a band of 50 and 25 kD characteristic of the molecular weight of hFab′ and light chain respectively. An immunoblot was detected with anti-human Fab′ and produced identical bands at 50 and 25 kD consistent with the presence of hFab′.

Cumulative urine output of radioiodine was assessed for one patient by collection of total urine. The majority of ^131^I-hDFM was eliminated via the urine in the first 24 h, equivalent to 20.2% of the injected activity at 23 h post collection; after which cumulative output decreased over time (37% i.a. at 47 h, and 41% i.a. at 60 h).

### Quality control

Radiolabelling of 10 individual patient aliquots of hDFM with ^131^I using the chloramine T method resulted in 85–92.5% labelling efficiency. Following removal of free iodine using a PD-10 column TLC analysis revealed 97–99% incorporation to the antibody.

Samples of each radioconjugate were routinely tested for immunoreactivity using a CEA antigen column. Results showed that 89–94% bound to the column compared to a non-CEA antibody control radiolabelled with ^131^I which bound non-specifically 2% of total recovered counts.

### Imaging results

^131^I-hDFM localised to 11 out of 14 of the known sites of tumour recurrence in eight patients known to have metastatic disease by standard radiological techniques. In the remaining patients (7 and 10) a true negative result (i.e. confirmation of no tumour) was observed which served as normal controls. For patient 2 a previously unidentified perineal lesion was discovered by imaging with ^131^I-hDFM, the recurrence was later confirmed by an MRI scan. A summary of the results is shown in Table 1

**Table 1:**
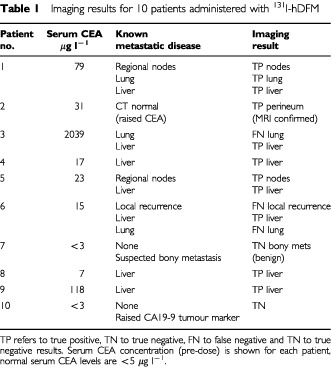
Imaging results for 10 patients administered with ^131^I-hDFM

. The sensitivity and specificity of ^131^I-hDFM as an imaging agent for the 10 patients in this study was 79 and 100% respectively.

**Figure 3:**
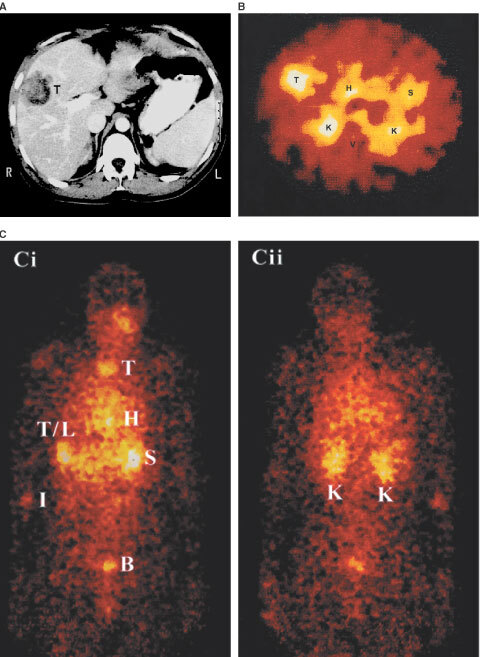
Imaging results for patient 5 24 h after injection of ^131^I-hDFM. (**A**) CT scan through the liver of where a liver metastasis is arrowed, (**B**) corresponding gamma camera cross-sectional SPECT image showing localisation of ^131^I-hDFM to the liver metastasis and (**C**) corresponding whole body image (i) anterior and (ii) posterior views. High amounts of radioactivity are shown in areas of intense colour (white–yellow). T/L=tumour/liver, H=heart, K=kidney, S=stomach, V=vertebra, B=bladder, I=injection site, T=thyroid.

An example of a SPECT image and corresponding CT scan is illustrated in Figure 3

demonstrating positive localisation of ^131^I-hDFM to a liver lesion. There is high activity in the kidneys due to clearance as shown in the whole body image (Figure 3Cii) and there is also evidence of unconjugated ^131^I manifested by the increased activity in the stomach and upper abdomen.

### Biodistribution

**Figure 4:**
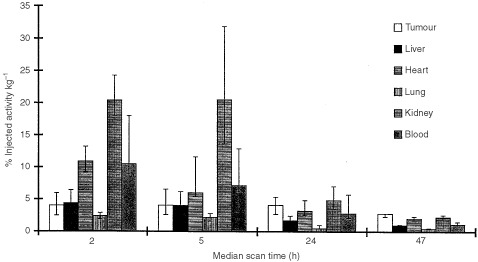
Biodistribution in main organs of ^131^I hDFM in the 10 study patients (eight for tumour). The bar represents the median value at each scan time. The error bars reflect 60% of the data values (75% for tumour).

Quantitative assessment of tumour and measurable normal tissue uptake of antibody by SPECT at each imaging time is shown in Figure 4

**Figure 5:**
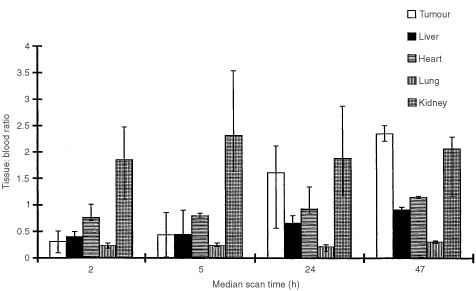
Tumour and organ to blood ratios of hDFM in 10 study patients (eight for tumour and 10 for other organs). The bar represents the median value at each scan time. The error bars reflect 60% of the data values (75% for tumour).

. ^131^I-hDFM localised to the tumour within 2 h of injection (median 4.0% i.a. kg^−1^) and tumour uptake reached a maximum median level of 4.1% i.a. kg^−1^ 5 h after injection. By 47 h the activity levels were higher in the tumour than in any other tissue which is illustrated more clearly in Figure 5

, where a tumour: blood ratio of 2.35 : 1 is observed. However, due to the wide variation of per cent uptake levels in tissues for all patients, differences in absolute uptake in different organs were not statistically significant. Four patients were imaged at 72 h post injection, and further patient scans at this time point were not performed because the low absolute level of activity remaining in the body meant that gamma scanning would be of limited value for dosimetric calculations.

The highest per cent uptake was in the kidney, where a level of up to 20.5% i.a. kg^−1^ was observed, indicating that a substantial part of kidney activity is due to excretion of ^131^I-hDFM in urine. This was significantly greater than for all other tissues at 2 and 5 h after injection (*P*<0.05 by Mann–Whitney *U*-test). By 24 h median levels had fallen to <5% of the injected activity kg^−1^, suggesting that most clearance occurs on the day of injection.

The levels of activity in the heart were similar at all time points to blood levels reflecting clearance of antibody from the circulation. There was no evidence of non-specific uptake of ^131^I-hDFM in other normal organs with measurable activity. No toxicity attribatable to the administration of hDFM was observed for the 10 patients in this study as assessed by the Common Toxicity Criteria (NCI CTC, 1988).

### Dosimetric analysis

The total residence time or area under the curve (AUC) of ^131^I-hDFM in various tissues was calculated from the biodistribution data. This data is expressed as tissue : blood ratios shown in Table 2

**Table 2:**
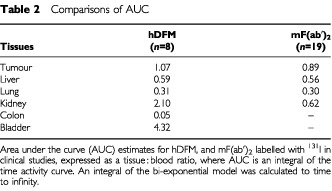
Comparisons of AUC

. Similar AUC ratios for ^131^I-mF(ab′)_2_ have also been evaluated from a previous clinical study for comparative purposes (unpublished data).

Tumour : blood ratios for hDFM were higher than for mF(ab′)_2_. Normal tissue distribution was similar except for kidney levels which were three-fold higher for hDFM compared to mF(ab′)_2_. Levels of activity in the liver, reflecting hepatic clearance, are comparatively low for both hDFM and mF(ab′)_2_.

### MIRD dosimetry

An assessment of toxicity to normal tissues was measured by estimating the total absorbed dose of ^131^I using the MIRD schema. Total estimates of absorbed dose are shown in Table 3

**Table 3:**
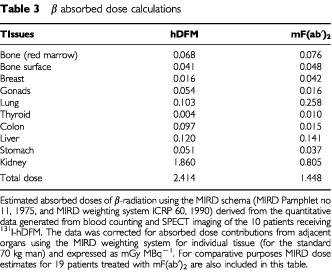
β absorbed dose calculations

. Dose to the bone marrow per MBq injected for ^131^I-hDFM was similar to that of ^131^I-mF(ab′)_2_. However, total absorbed dose was significantly greater for ^131^I-hDFM compared to ^131^I-mF(ab′)_2_, and this was due to the high contribution from the kidney dose. Total dose for ^131^I-hDFM was 1.67-fold greater than for ^131^I-mF(ab′)_2_. This relatively high dose to the kidneys on administration of ^131^I-hDFM could therefore limit the dose that may be administered therapeutically.

### Pharmacokinetics

The stability of ^131^I-hDFM was assessed by analysis of plasma samples by HPLC for the presence of immune complexes and breakdown products. Low levels of breakdown products at lower molecular weight fractions were observed for all patients and increased as a per cent of the total recovered counts over time (data not shown). The calibration markers revealed that the molecular weight of this peak was <1.3 kDa, and it is therefore likely that this peak is free iodine or iodo-tyrosine, which is released from antibody over time. At 10 min post injection 2–5% of recovered activity eluted in the low molecular weight (<1.5 kDa) fraction and this increased over time to 18–26% at 72 h.

Low levels of aggregates, in the form of radioactivity appearing in high molecular weight fractions (void volume), was observed for some patients at later time points. This appeared to be related to the amount of CEA antigen in the circulation measured by serum ELISA. For two patients with relatively low levels of serum CEA (⩽10 μg ml^−1^) total aggregate formation to 26 h was ⩽3.2% and ⩽7.5% up to 72 h. For seven patients with intermediate levels of serum CEA (15–79 μg ml^−1^), aggregate formation to 24 h of ⩽11.9% and ⩽23.3% to 72 h was observed. For one patient (3) with a very high serum CEA level (2039 μg ml^−1^), a large amount of aggregate formation was observed. Total aggregate formation to 24 h of 29% was observed which decreased slightly over time to 72 h (16.4%). Overall clearance of the hDFM peak for patient 3 was slightly faster than for most of the other patients but similar to patient 1, who had the third highest level of CEA (79 μg ml^−1^). It is therefore likely that these aggregates were in the form of immune complexes consisting of serum CEA and hDFM, which resulted in faster clearance of antibody from the circulation.

Plasma samples were also analysed for binding to CEA coated ELISA plates as a measure of immunoreactivity. Positive binding (CPM) to CEA compared to control PBS wells was observed for all samples on the day of administration. However, since a large amount of antibody was cleared from the circulation in the first 24 h, the dilution factor was too great to measure binding on subsequent days post administration.

Pre-treatment and post treatment (>14 day) serum samples were routinely collected to monitor immunogenicity. There was an increase in both IgM and IgG HAHA titre for patient 3, and there was evidence of a minor response in IgM titre for patient 5. There was no evidence of IgM or IgG response in the remaining eight patients in this study. Patient 3 was known to have a very high level of circulating CEA, and formation of immune complexes (>200 kD) was observed by HPLC within 10 min of antibody administration.

## DISCUSSION

A combination of molecular and chemical cross-linking technologies have been employed here to generate a new antibody construct that has some advantages over conventional intact antibodies and their fragments. This potentially less immunogenic humanised version of A5B7 hFab′ was the first recombinant and cross-linked form of A5B7 to be administered to patients. In addition this product was also one of a very limited number of antibodies to be produced in bacteria for the clinic (Begent *et al*, 1996).

It is a requirement of any regulatory organisation that new drugs to be administered for phase I/II clinical studies be thoroughly characterised and analysed for safety. The cell line, production and purification processes for A5B7 hDFM were validated and after full characterisation and toxicity studies the clinical batch was deemed suitable for patient administration (Begent *et al*, 1993). One of the major advantages of antibody production in bacteria is the non-requirement for extensive testing for viruses and mammalian DNA, which is expensive and time consuming.

Storage conditions are important to assess prior to clinical use. However, one of the problems associated with any clinical study is the length of time allocated to this. Short-term stability studies for A5B7 hDFM were performed, but longer-term stability (1 year) revealed that storage conditions (PBS pH 7.4, 4°C) were sub-optimal and resulted in some breakdown to hFab′ (approximately 10%). Extensive stability studies of hDFM in various buffers at different temperatures are a necessary requirement before further clinical studies with this antibody are planned. Recent stability studies have shown improved stability of hDFM when stored in a lower pH buffer (pH 5.5–6) or in a lyophilised form.

In this pilot imaging study of 10 patients injected with ^131^I-hDFM, at least one known tumour site was localised in all patients, with tumour recurrence shown by conventional imaging techniques. This provided clinically useful information in either confirming patients had recurrent disease, identifying a new lesion (one patient), or confirming there was no disease (two patients). The three false negative findings were in two patients. In one patient a lung metastasis and local recurrence and in the other a lung metastasis, were not disclosed by antibody imaging but were seen by CT.

Blood clearance kinetics defined in all 10 patients demonstrated similar blood clearance and half-lives to mF(ab′)_2_ in man. This was surprising as direct comparisons for murine parent antibodies and engineered chimeric or humanised versions of the same antibody have frequently resulted in prolonged half-lives (LoBuglio *et al*, 1989). However, a direct comparison of humanised antibody fragments with the murine equivalent has not been carried out previously. Also half-life is known to be dependent on protein dose, resulting in an increased clearance rate of lower doses of the same antibody (Stephens *et al*, 1995). This may be particularly relevant when circulating antigen may be present as in this case. A relatively high dose of A5B7 mF(ab′)_2_ (10 mg per patient) was administered to patients for RIT compared to the imaging dose of 0.5 mg hDFM administered in this study. Therefore a longer circulating half-life may be observed for higher therapy doses of hDFM.

Some studies have attempted to address the relationship of circulating antigen and half-life (Davidson *et al*, 1991; Pimm, 1995; Behr *et al*, 1996). In one study, data from 275 patients was analysed, and results implied that antibodies administered to patients with normal plasma CEA (<5 μg l^−1^) had longer half-lives than in patients with CEA levels in excess of 10 μg l^−1^ (Behr, 1996). In this study only two patients had CEA levels <5 μg l^−1^ (patients 7 and 10) and their plasma or blood half-life was not significantly different to the other patients. However for one patient (3) with very high levels of CEA (2039 μg l^−1^) formation of antibody complexes was observed by HPLC soon after antibody administration. This resulted in faster clearance of hDFM from the circulation and a corresponding shorter β-half-life in blood of 14 h compared to the median of 20.3 h. Low tumour uptake levels, especially at early time points (2 h : 0.9% i.a. kg^−1^, median value 4%), may have been the result of more rapid clearance of hDFM from the circulation. Despite this however, positive tumour imaging was reported.

The biodistribution of ^131^I-hDFM was extensively studied by gamma camera imaging at various scanning times to enable quantification of absorbed dose to radiosensitive organs. The results were compared to similar clinical data using ^131^I-mF(ab′)_2_ (Lane *et al*, 1994) in an attempt to assess whether ^131^I-hDFM would be a better candidate for RIT studies. AUC (Table 2) estimates revealed a higher overall tumour to blood ratio for hDFM (1.07) compared to mF(ab′)_2_ (0.89) which is desirable for RIT.

AUC (Table 2) and MIRD (Table 3) estimates indicated a similar biodistribution and clearance of both fragments except for high kidney uptake of ^131^I-hDFM. In nude mice experiments we have observed similar biodistribution patterns including kidney uptake for ^131^I-labelled hDFM, mDFM and mF(ab′)_2_, implying that this high kidney uptake of ^131^I-hDFM was specific only to man.

It is unclear why there are major differences in kidney uptake of hDFM and mF(ab′)_2_; the altered clearance pattern of similar sized molecules suggests that other factors such as shape and charge may also influence the rate of clearance via the kidney (Sumpio and Hayslett, 1985). The presence of the thio-ether bond could in some way affect the clearance of hDFM leading to non-specific uptake by the renal tubules. High stability of the thio-ether bond could result in slower catabolism resulting in a longer kidney residence time. We observed breakdown of hDFM to hFab′ occurring in the kidney's as analysed by SDS–PAGE and autoradiography of urine samples, indicating renal catabolism occurs. Further experiments were performed to assess whether hDFM cross-reacted with human kidney samples from four individuals, by performing immunohistochemistry experiments. These findings showed no non-specific binding of hDFM to normal human kidney, indicating there was no binding to a renal antigen.

Dosimetry estimates indicate that the dose-limiting organ for hDFM is the kidney, and if hDFM is to be considered for RIT it would obviously be desirable to reduce kidney levels of activity. A promising option is to attach polyethylene glycol (PEG) to hDFM which in a recent study in nude mice bearing human colorectal tumour xenografts was shown to increase the circulatory half-life resulting in higher tumour uptake levels and a lower absorbed dose to the kidney (Casey *et al*, 2000).

The red bone marrow is highly radiosensitive and myelosuppression often limits the total dose that may be administered therapeutically (Siegel *et al*, 1990). It is therefore important to assess the toxicity to bone marrow and other radiosensitive organs, such as the kidney, in low-dose clinical studies such as the one described here, to predict possible toxicity on administration of higher doses. Dose to the red bone marrow is usually assumed to be proportional to the blood dose, where marrow dose is equivalent to blood dose×0.4 (Siegel *et al*, 1990). The maximum tolerated doses (MTD) for radiosensitive organs such as red bone marrow and kidney have been evaluated by conventional radiotherapy studies to be <200 and <1500 cGy respectively (Fawwaz *et al*, 1986). Therefore, by considering these limits, the maximum tolerated activity can be estimated using the MIRD dosimetry calculations for ^131^I-hDFM and ^131^I-mF(ab′)_2_ illustrated in Table 3. The MTD that allows for maximum kidney tolerance would be: 1500/0.186=8.06 GBq for ^131^I-hDFM, and 1500/0.0805=18.6 GBq for ^131^I-mF(ab′)_2_; and for maximum tolerance to the red marrow and bone surface: 200/0.0109=18.4 GBq for ^131^I-hDFM, and 200/0.0124=16.1 GBq for ^131^I-mF(ab′)_2_. This implies that the dose limiting organ for hDFM is the kidney and the bone marrow for mF(ab′)_2_. However, these are estimated doses, and an organised dose escalation study involving detailed toxicity assessment is required before a true MTD is obtained. The MTD for A5B7 ^131^I-mF(ab′)_2_ has previously been shown to be approximately 3.29 GBq m^2^ (Lane *et al*, 1994), implying that the MIRD weighting for red marrow is underestimated, as toxicity was observed at much lower levels than was anticipated using the MIRD estimates.

Although there is at present little data to suggest renal damage after RIT, impairment of renal function has been observed with whole abdominal irradiation in patients given doses >20 Gy. However with similar or higher doses administered in a fractionated manner there has been no evidence of renal toxicity up to 5 years after treatment (Irwin *et al*, 1996). Although the toxicity levels of external whole body irradiation and RIT cannot be directly compared, this implies that a fractionated approach to administration of hDFM may also be worth considering.

In the present study only one patient elicited a positive immune response 2 weeks after ^131^I-A5B7 hDFM injection. However, this patient also had a level of high circulating CEA and evidence of immune complexes soon after antibody injection. Immune complexes may cause an increase in the propensity to produce anti-antibodies due to increased recognition by cells of the immune system. This is effectively similar to the natural host defence mechanism by the immune system on attack by a foreign antigen when host antibodies complex with antigen and present the antigen as foreign to cells of the immune system (Roitt, 1991). A similar mechanism may occur here when circulating host antigen complexes with foreign antibody.

There have been only a few reports describing the immunogenicity of humanised antibodies in clinical studies, and as yet no studies involving humanised fragments. Hale *et al* (1988) reported that lymphoma patients treated with multiple doses of this antibody showed no antibody response to a humanised version of CAMPATH-1H. In a further study CAMPATH-1H was administered to rheumatoid arthritis patients repeatedly over 10 days. No immune response was reported following this first course of treatment but following the second course of treatment three out of four patients showed a detectable immune response. However this immune response was not characterised. In another study the humanised antibody CDP571 was administered as a single dose ranging from 0.1–10 mg kg^−1^ to human volunteers (Stephens *et al*, 1995). At low doses a weak immune response of IgM anti-idiotype was detectable and at higher doses responses were lower or undetectable. In a pilot imaging study none of the four patients with B-cell lymphomas receiving 2 mg of the humanised LL2 antibody developed an immune response (Juweid *et al*, 1995). In a further small study, eight patients received the anti-CEA humanised hMN-14 antibody (0.5–20 mg) and the immune response was measured in five patients (Sharkey *et al*, 1995). Four of these patients received 2–3 injections and two received high protein doses (10–20 mg). An immune response was not recorded for any of these patients during the 4–5 week follow up period.

These initial results suggest that humanised antibodies are less immunogenic than murine antibodies, especially at higher concentrations, and therefore should enable repeated administration. However, all of the above studies, including the one described here, involved small numbers of patients, low doses and usually only one injection of humanised antibody. Further analysis is necessary to study the immune response of humanised antibodies and fragments in larger clinical studies. If hDFM is to be considered for RIT, studies must be performed to determine whether the immunogenicity of hDFM will remain low at higher doses and after multiple injections. Furthermore the potential immunogenicity of the maleimide linker is a significant issue. Clearly, in future clinical studies involving higher doses of antibodies or fragments containing the maleimide cross-linker characterisation of the immune response is essential. There have to date been no reported studies to indicate that the maleimide linker is immunogenic in animals or patients.

It is our aim to design an antibody construct based on the IgG A5B7, which has optimal properties for RIT. We have shown in this pilot clinical study that A5B7 ^131^I-hDFM proved to be effective in targeting tumours in all patients with known lesions with a median maximum of 4.1% of the total injected activity. The area under the curve data revealed a higher estimated tumour to blood ratio for A5B7 hDFM compared to A5B7 mF(ab′)_2_ implying it will be superior for RIT. The only problem that did not become evident until this clinical trial was performed was the high level of kidney uptake in patients. We have further optimised DFM by the addition of PEG, which in our nude mouse model showed superior tumour uptake levels and lower kidney toxicity (Casey *et al*, 2000).

In conclusion, we have demonstrated that it is possible to engineer superior antibody products for optimal tumour targeting. The clinical study revealed improved tumour localisation and lower immunogenicity for hDFM compared to mF(ab′)_2_; therefore indicating that hDFM should prove to be our optimal form of A5B7 anti-CEA antibody for future RIT.

## References

[bib1] AdairJABodmerMWMountainAOwensRJ1992 International patent application. CDR grafted anti-CEA antibodies and their production. WO 92/01059

[bib2] BegentRHJChesterKAConnorsTCrowtherDFoxBGriffithsEHinceTALedermanJAMcVieJGMinorPSecherDSSchwartsmannGThorpeRWilbinCZwierzinaH1993 Cancer Research Campaign Operation Manual for control recommendations for products derived from recombinant DNA technology prepared for investigational administration to patients with cancer in phase I trials Eur J Cancer 29A 19071910 8260253 10.1016/0959-8049(93)90549-u

[bib3] BegentRHJVerhaarMJChesterKACaseyJLGreenAJNapierMPHope-StoneLCushenNKeepPAJohnsonCJHawkinsREHilsonAJWRobsonL1996 Clinical evidence of efficient tumour targeting based on single-chain Fv antibody selected from a combinatorial library Nature Medicine 2 979984 10.1038/nm0996-979 8782454

[bib4] BehrTMSharkeyRMJuweidMIDunnRMZhiliangYZhangCHSiegelJAGoldDVGoldenbergDM1996 Factors influencing the pharmacokinetics, dosimetry and diagnostic accuracy of radioimmunodetection and radioimmunotherapy of carcinoembryonic antigen-expressing tumours Cancer Res 56 18051816 8620497

[bib5] BorrebaeckCAKDanielssonLOhlinMCarlssonJCarlssonR1990 The use of in vitro immunisation cloning of variable regions and SCID mice for the production of human monoclonal antibodiesInTherapeutic monoclonal antibodiespp115UK: Stockton Press

[bib6] CaseyJLKeepPAChesterKARobsonLHawkinsREBegentRHJ1995 Purification of bacterially expressed single chain Fv antibodies for clinical applications using metal chelate chromatography J Immunol Methods 179 105116 7868918 10.1016/0022-1759(94)00278-5

[bib7] CaseyJLKingDHJChaplinLCHainesAMRPedleyRBMountainAYarrantonGTBegentRHJ1996 Preparation, characterisation and tumour targeting of cross-linked divalent and trivalent anti-tumour Fab′ fragments Br J Cancer 74 13971405 8912535 10.1038/bjc.1996.555 PMC2074792

[bib8] CaseyJLPedleyRBKingDJBodenRChapmanAPYarrantonGTBegentRHJ2000 Improved tumour targeting of di-Fab′ fragments modified with polyethylene glycol Tumour Targeting 4 235244

[bib9] DavidsonBRBabichJYoungHWaddingtonWClarkeGShortMBoulosPStylesJDeanC1991 The effect of circulating antigen and radiolabel stability on the biodistribution of an indium labelled antibody Br J Cancer 64 850856 1931605 10.1038/bjc.1991.412 PMC1977445

[bib10] EmerySCAdairJR1994 Humanised monoclonal antibodies for therapeutic applications Exp Opin Invest Drugs 3 241251

[bib11] FawwazRAWangTSTSrivastavaSCHardyMA1986 The use of radionuclides for tumour therapy Nucl Med Biol 13 429436

[bib12] GreenAJDewhurstSEBegentRHJBagshaweKDRiggsSJ1990 Accurate quantification of ^131^I distribution by gamma camera imaging Eur J Nucl Med 16 361365 2351184 10.1007/BF00842793

[bib13] HaleGDyerMJClarkeMR1988 Remission induction in non-Hodkin lymphoma with reshaped human monoclonal antibody Campath-1H Lancetii 13941396 10.1016/s0140-6736(88)90588-0 2904526

[bib14] HaleGPhillipsJM1995 Clinical trials with CAMPATH-1 and other monoclonal antibodies Biochem Soc Transactions 23 10571063 10.1042/bst0231057 8654681

[bib15] IrwinCFylesAWongCSCheungCMZhuY1996 Late renal function following whole abdominal irradiation Radiother and Oncol 38 257261 10.1016/0167-8140(95)01702-x 8693108

[bib16] JuweidMSharkeyRMMarkowowitzABehrTSwayneLCDunnRHansenHJSheivitzJLeungSORubinADHerskovicTHanleyDGoldenbergDM1995 Treatment of non-Hodgkins lymphoma with radiolabeled murine, chimeric or humanised LL2, an anti CD22 monoclonal antibody Cancer Res 55Suppl58995907 7493367

[bib17] LaneDMEagleKFBegentRHJHope-StoneLDGreenAJCaseyJLKeepPAKellyAMBLedermannJAGlaserMGHilsonAJW1994 Radioimmunotherapy of metastatic colorectal tumours with iodine-131-labelled antibody to carcinoembryonic antigen: phase I/II study with comparative biodistribution of intact and F(ab′)_2_ antibodies Br J Cancer 70 521525 8080740 10.1038/bjc.1994.338 PMC2033373

[bib18] LedermannJABegentRHJBagshaweKDRiggsSJSearleFGlaserMGGreenAJDaleRG1988 Repeated antitumour antibody therapy in man with suppression of the host response by Cyclosporin A Br J Cancer 58 654657 3265333 10.1038/bjc.1988.279 PMC2246821

[bib19] LoBuglioAFWheelerTHTrangJHaynesARogersKHarveyEBSunLGhrayebJKhazaeliMB1989 Mouse/human chimaeric monoclonal antibody in man: Kinetics and immune response Proc Natl Acad Sci USA 86 42204224 2726771 10.1073/pnas.86.11.4220 PMC287422

[bib20] LonbergNTaylorLDHardingFATrounstineMHigginsKMSchrammSRKuoCCMashayekhRWymoreKMcCabeJGMunoz-O'ReganDO'DonnellSLLapachetSGBengoecheaTFishwildDMCarmackCEKayRMHuszarD1994 Antigen-specific human antibodies from mice comprising four distinct genetic modifications Nature 368 856859 8159246 10.1038/368856a0

[bib21] Operation Manual for control of production, pre-clinical toxicology and phase I trials of anti-tumour antibodies and drug antibody conjugates1986 Br J Cancer 54 577578 10.1038/bjc.1986.209 PMC2001634 3756089

[bib22] PimmMV1995 Circulating antigen: Bad or good for immunoscintigraphy? Nucl Med Biol 22 137145 7767306 10.1016/0969-8051(94)00098-5

[bib23] RoittI1991 Essential ImmunologyBlackwell Scientific publications

[bib24] SharkeyRMJuweidMShevitzJBehrTDunnRSwayneLCWongGYBlumenthalRDGriffithsGLSiegelJALeungSHansenHJGoldenbergDM1995 Evaluation of a complementarity-determining region grafted (humanised) anti-carcinoembryonic antigen monoclonal antibody in preclinical and clinical studies Cancer Res 55Suppl59355945 7493374

[bib25] SiegelJAWesselsBWWatsonEEStabinMGVriesendorpHMBradleyEWBadgerCCBrillABKwokCSSticklyDREckermanKFFisherDRBuchsbaumDJOrderSE1990 Bone marrow dosimetry and toxicity for radioimmunotherapy Antibody Immunoconj Radiopharm 3 213233

[bib26] StephensSEmtageSVetterleinOChaplinLBebbingtonCNesbittASopwithMAthwalDNpvakCBodmerM1995 Comprehensive pharmacokinetics of a humanised antibody and analysis of residual anti-idiotytic responses Immunology 85 668674 7558164 PMC1383798

[bib27] SumpioBEHayslettJP1985 Renal handling of proteins in normal and disease states Quart J Med 57 611635 4080952

[bib28] TheodorouNA1995 Radioimmunoguided surgery for colorectal cancerInNew antibody technology and the emergence of useful cancer therapyBegent RHJ, Hamblin A (eds), pp 55. Proceedings of the Tufton Trust conference at Royal Society of MedicineLondon: RSM Press

[bib29] VerhoeyenMMilsteinCWinterG1988 Reshaping human antibodies: Grafting an antilysozyme activity Science 239 15341536 2451287 10.1126/science.2451287

[bib30] WinterGGriffithsADHawkinsREHoogenboomHR1994 Making antibodies by phage display technology Ann Rev Immunol 12 433455 8011287 10.1146/annurev.iy.12.040194.002245

